# Model-Agnostic Binary Patch Grouping for Bone Marrow Whole Slide Image Representation

**DOI:** 10.1016/j.ajpath.2024.01.012

**Published:** 2024-02-05

**Authors:** Youqing Mu, Hamid R. Tizhoosh, Taher Dehkharghanian, Saghir Alfasly, Clinton J.V. Campbell

**Affiliations:** ∗ Department of Mechanical and Industrial Engineering, University of Toronto, Toronto, Ontario, Canada; † Department of Pathology and Molecular Medicine, McMaster University, Hamilton, Ontario, Canada; ‡ Rhazes Lab, Artificial Intelligence and Informatics, Mayo Clinic, Rochester, Minnesota; § Department of Nephrology, University Health Network, Toronto, Ontario, Canada

## Abstract

Histopathology is the reference standard for pathology diagnosis, and has evolved with the digitization of glass slides [ie, whole slide images (WSIs)]. While trained histopathologists are able to diagnose diseases by examining WSIs visually, this process is time consuming and prone to variability. To address these issues, artificial intelligence models are being developed to generate slide-level representations of WSIs, summarizing the entire slide as a single vector. This enables various computational pathology applications, including interslide search, multimodal training, and slide-level classification. Achieving expressive and robust slide-level representations hinges on patch feature extraction and aggregation steps. This study proposed an additional binary patch grouping (BPG) step, a plugin that can be integrated into various slide-level representation pipelines, to enhance the quality of slide-level representation in bone marrow histopathology. BPG excludes patches with less clinical relevance through minimal interaction with the pathologist; a one-time human intervention for the entire process. This study further investigated domain-general versus domain-specific feature extraction models based on convolution and attention and examined two different feature aggregation methods, with and without BPG, showing BPG's generalizability. The results showed that using BPG boosts the performance of WSI retrieval (mean average precision at 10) by 4% and improves WSI classification (weighted-F1) by 5% compared to not using BPG. Additionally, domain-general large models and parameterized pooling produced the best-quality slide-level representations.

Histopathology is the extraction of microscopic information from preserved sections of human tissue by a pathologist, providing information about the biological state of a patient. It is considered the reference standard method for supporting a diagnosis in pathology.^1^ Digitizing tissue glass slides into whole slide images (WSIs), known as digital pathology, has revolutionized computational pathology techniques, enabling the efficient processing and storage of large amounts of histopathology data.^2^

Trained histopathologists can make an accurate tissue assessment by visually inspecting WSIs on high-resolution settings. However, the large spatial dimensionality of WSIs makes analyzing these images a time-consuming and inefficient process. In response to this challenge, scientists in this domain have embraced automated analysis of WSIs as a means to quantify pertinent anatomic structures. Deep learning models (ie, deep neural networks) have emerged as a promising solution, providing state-of-the-art performance in a wide variety of tasks, including tumor grading, tumor segmentation, and disease prediction.^3^, ^4^, ^5^, ^6^ However, despite the potency of deep learning, several challenges persist. The lack of labeled WSI data, heterogeneity in images, and complexity of features all pose technical difficulties that need to be overcome to achieve reliable results.^7^ Thus, pipelines are designed to address these issues and to make deep learning models more practical for the histology-specific domain.^8^

Previous studies involving WSI processing and analysis focused primarily on patch-level classification, segmentation, tumor area detection, and similar tasks.^9^, ^10^, ^11^, ^12^ Recently, slide-level representation learning has gained more attention.^13^, ^14^, ^15^, ^16^ Slide-level representation refers to deriving information from the entire WSI (ie, from all or many of its patches) into a single feature vector capturing collective WSI morphologic semantics. Similar to word2vec,^17^ compact vector representations of WSIs can enable a wide range of computational pathology applications, such as interslide search, multimodal training, slide-level classification, and survival prediction.^18^, ^19^, ^20^, ^21^

A typical pipeline of slide-level representation starts with tiling the WSIs into arbitrarily defined smaller subimages known as patches. Then, a subset of patches is selected through a sampling procedure. The selection can be performed randomly, stain-based, and spatial clustering,^19^ or based on certain criteria, such as using expert pathologist–annotated regions of interest (ROIs), or by pixel values. Next, deep features are extracted from each patch. These features are derived predominantly from pretrained deep learning models, such as DenseNet^22^ and ResNet.^23^ Finally, the WSI-level representation features are derived from the patch-level features via different aggregation methods such as max pooling, average pooling, attention pooling, deep generative modeling, and the Fisher vector.^16^^,^^24^ The choice of the methods and parameters in each step of the pipeline can affect the quality of the final representation. Thus, optimizing the steps of this pipeline is an ongoing research area in digital pathology.

To improve the quality of the final slide-level representation, current studies focus mainly on optimizing patch sampling, feature extraction, and aggregation steps.^4^^,^^21^^,^^24^, ^25^, ^26^ However, despite the importance of these steps, they may not be sufficient to optimize the quality of the final slide-level representation. Specifically, a step between patch feature extraction and aggregation steps to filter out irrelevant, redundant, and noisy patches (low informational content) is generally ignored.

We therefore hypothesized that a simple, pathologist-driven improvement stage between patch feature extraction and aggregation may enhance the final quality of slide-level representations. Specifically, bone marrow histopathology images were studied as a test case given the relatively clear delineation between tissue regions (diagnostically relevant) and nontissue regions (bone or adipose tissue, generally nondiagnostically relevant). This relatively simple and almost binary separation between regions provides a useful test case for pathologist-driven augmentation of WSI representation quality. Hence, the proposed binary patch grouping (BPG) step could act as an automatic ROI selection based on patch features, effectively filtering out irrelevant feature vectors to enhance representation quality. This could be achieved by using clustering techniques to group clearly distinct patch features and keeping only the relevant groups for aggregation. Rather than replacing state-of-the-art pipelines, this method functions as an enhancement module that can be incorporated into different pipelines, and involves minimal but important expert pathologist input.

The k-means is a widely used unsupervised clustering algorithm that can group similar features together effectively.^27^^,^^28^ It has been applied successfully for WSI processing in various steps, such as patch sampling, patch clustering, feature grouping, and WSI visualization.^15^^,^^19^^,^^29^, ^30^, ^31^ A hypothesis for this study was that applying the k-means algorithm with a *k* = 2, resulting in a partition of *n* data points into two clusters, could help eliminate irrelevant or less-significant patches. This avoids reliance on tedious manual patch-level labels, and instead requires a pathologist to examine and decide which cluster is preferred. This k-means–driven BPG method is a one-time task for the entire model design/training and inference process, with a negligible burden on the pathologist.

Using BPG to remove diagnostically irrelevant (or less relevant) patches was intended to improve slide-level representation quality. To validate this hypothesis experimentally, 633 WSIs (described in Data Set) were used to evaluate the effect of the BPG step in the pipeline. The evaluation covered various published feature extractors and aggregation methods used in WSI representation to investigate the effectiveness of domain-general versus domain-specific models, as well as transformer-based versus convolution-based models. This study found that domain-general attention models (trained with ImageNet via distillation with no supervision^32^) in combination with BPG and parameterized pooling produced the best-quality slide-level representations. Additionally, domain-specific models (both attention- and convolution-based) that have been trained with histopathology images were inferior to some domain-general models (that only trained on natural nonhistology images), which was an unexpected finding.

## Materials and Methods

### Data Set

The 717 bone marrow trephine biopsy WSIs (belonging to 616 patients) were deidentified retrospectively and annotated with only a synopsis, spanning a period of 1 year. Glass slides were scanned at ×40 magnification. The data comprised a full range of diagnoses at a large hematology reference center throughout this time period. The study was conducted under Hamilton Integrated Research Ethics Board study protocol 7766-C.

Patches (512 × 512 pixels) were extracted at a ×40 magnification level. Patches that were deemed mainly blank based on their RGB values were excluded. Additionally, patches with a file size smaller than 80 KB were eliminated because they typically do not contain tissue fragments.

Of these, 673 WSIs with more than 64 extracted patches were selected for further analysis. Because WSIs with fewer than 64 patches were likely to be derived from poor-quality original glass slides, the remaining 44 WSIs with fewer than 64 patches were disregarded.

WSI-level labels were generated by simplifying the predictions of a fine-tuned Bidirectional Encoder Representations from Transformers language model on WSI synopses, as previously reported by Mu et al.^33^ The Bidirectional Encoder Representations from Transformers language model predictions took the form of a multilabel task. In this work, these predictions were simplified to broad diagnostic categories using the following rules.

• If normal is in a multilabel prediction, this prediction will be simplified as normal.
• If any label is acute leukemia related, this prediction will be simplified as acute leukemia (eg, acute myeloid leukemia; hypercellular becomes acute leukemia).
• If any label is myelodysplastic syndrome related, this prediction will be simplified as myelodysplastic syndrome (eg, hypercellular; myelodysplastic syndrome becomes myelodysplastic syndrome).
• If any label is plasma cell neoplasm related, this prediction will be simplified as plasma cell neoplasm (eg, hypercellular; plasma cell neoplasm becomes plasma cell neoplasm).
• If any label is lymphoproliferative disorder related, this prediction will be simplified as lymphoproliferative disorder (eg, hypercellular; lymphoproliferative disorder becomes lymphoproliferative disorder).
• If any label is myeloproliferative neoplasm related, this prediction will be simplified as lymphoproliferative disorder (eg, myeloproliferative neoplasm; fibrosis becomes myeloproliferative neoplasm).

The 40 WSIs that did not meet the previously mentioned criteria were discarded because their label groups had fewer than 10 WSIs, making it inadequate to generate new classes. As a result, a primary data set was generated consisting of 633 WSI and label pairings for experiments on a slide-level representation pipeline (Supplemental Table S1). For these experiments, five-repeat Monte Carlo cross-validation^34^ was performed. For each cross-validated fold, 633 WSIs were partitioned randomly into a training set (50% of cases; ie, 316 WSIs) and a test set (50% of cases; ie, 317 WSIs) and maintained the proportion of classes consistently in each set.

### Feature Extractors

The performance of the pipeline arguably is affected by the choice of domain-general (DG) versus domain-specific (DS) pretrained models as feature extractors. The main differences in the pretrained models are in their training data set and model complexity (ie, size). Typically, DG data sets are large and diverse data sets because it is easier to collect general data than specific data such as medical images. For instance, ImageNet, a general data set, comprises more than 1.2 million labeled images belonging to 1000 classes,^35^ whereas The Cancer Genome Atlas, a pathology-specific data set, comprises approximately 32,000 hematoxylin and eosin–stained images from more than 11,000 specimens belonging to 32 classes (subtypes).^36^ Previous studies have shown that DS models trained on domains closely related to the transfer-learning domain outperform DG models.^37^^,^^38^ However, large models trained on these relatively small DS data sets, compared with smaller models, have a higher probability of overfitting and being sensitive to input changes,^37^ even though empiric evidence suggests that larger models may outperform small models. However, as pretrained methods shift from supervised to self-supervised learning (SSL),^39^^,^^40^ this paradigm may no longer hold. Unlike supervised learning, SSL is designed to learn representations, which are less affected by domain differences.^41^ Thus, a DG large model trained with SSL potentially can outperform DS models in transfer learning because it produces richer features for the downstream model to learn and is less prone to overfitting thanks to its larger and more diverse training data sets.

The study used pretrained models from the two main deep network architectures, namely convolution and transformer, including DenseNet-121^22^ and KimiaNet^38^ to represent convolution in DG and DS models, respectively. In addition, the ViT-16/256 model from HIPT_4K (ViT-16/256)^42^ and the ViT-S/16 model from DINO^32^ were used to represent a transformer in DG and DS models, respectively. To distinguish the vision transformer models (ViT) between DINO's from HIPT_4K's, DINO's ViT was named DINO in this study. Patches (512 × 512 pixels) at ×40 magnification were resized to 256 × 256 pixels to align with the ViT-16/256 model's size and ×20 magnification input condition and DINO's input size condition.

DenseNet-121 and KimiaNet are convolutional neural network–based networks with the same architecture, whereas HIPT (ViT-16/256) and DINO are built on the transformer architecture.^43^ DenseNet-121 and KimiaNet have 7,978,856 parameters (relatively small), whereas HIPT (ViT-16/256) and DINO have 21,665,664 parameters (ie, 270% larger). Both KimiaNet and ViT-16/256 were pretrained on histopathology data (The Cancer Genome Atlas, DS), whereas DenseNet-121 and DINO were pretrained only on ImageNet (DG) without specific domain adaptation to histopathology images. By using these four different feature extractors, this study compared the performance of convolutional neural network and transformer models and the impact of DS versus DG feature extractors on the final results (ie, WSI representation quality). This experimental setting was designed to enable verification of the robustness and generalizability of this method, regardless of the feature extractor chosen.

### BPG

**Figure 1:**
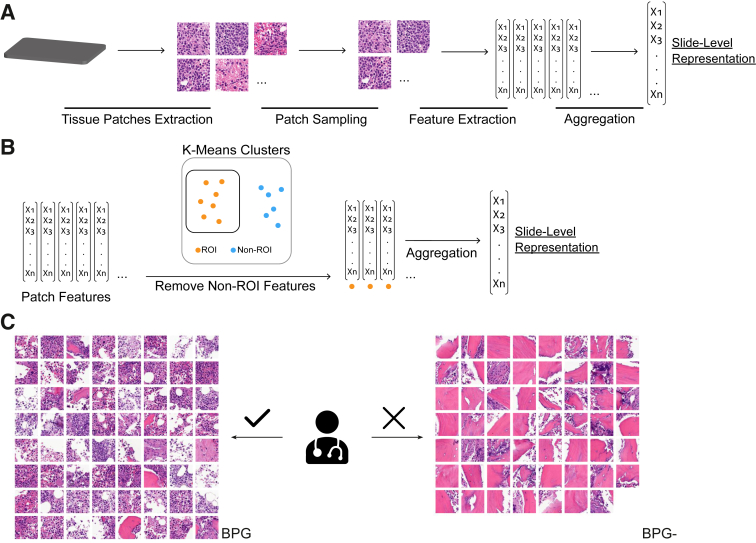
Slide-level representation pipeline. **A:** The pipeline of slide-level representation starts with the division of the whole slide image (WSI) into smaller regions known as patches. Some patches then are selected through sampling to extract deep features. Subsequently, patch features are aggregated to generate a single vector to serve as slide-level representation. **B:** The proposed method involves using binary patch grouping (BPG) before aggregating patch features to slide-level representation. This step filters out the less-relevant group of patches for the given WSI before aggregating them into a slide-level representation. **C:** The minimal pathologist intervention in the process is the marking of the cluster to be eliminated. Original magnification, ×40. ROI, region of interest.

BPG training was prepared by first randomly sampling *n* patches (ie, *n* feature vectors) per slide from all 717 scanned WSIs, except for one randomly chosen WSI that served as a reference. This preparation process of BPG is independent of the slide representation pipeline, so the removal criteria applied to WSIs in the Data Set section were not applied, and all 717 scanned WSIs were used. In the experiments, *n* = 96 was set empirically, and when a WSI had fewer than *n* patches, all of its patches were included without sampling. In total, a set of 64,790 patch feature vectors from 716 WSIs were sampled and was called the Profiling Data Set. Next, the k-means algorithm was used to cluster all the patch feature vectors in this data set into two groups. The k-means then were used on the patch feature vectors from the reference slide and a pathologist examined the two clusters, deciding one as the desired target and the other as the nontarget (the only pathologist intervention step) (Figure 1). The former cluster is regarded as the result of BPG and the latter as the result of negative BPG (BPG-). The labeling of reference clusters of one slide is the minimal expert interaction required to exploit the benefits of patch categorization.

### Aggregation Methods

In multiple-instance learning, aggregation refers to the process of aggregating the representations of individual instances into a single representation for the whole bag,^44^^,^^45^ which can be achieved by pooling methods. As an example, if a bag is a collection of instances, then the goal is to understand the characteristics of the entire bag.

Pooling can be divided into two categories: nonparameterized and parameterized pooling. Nonparameterized pooling methods do not have adjustable parameters and summarize information in a bag using fixed operations such as maximum taking or averaging over a set of inputs. They can be expressed as follows:

(1) $$f_{\text{non}-\text{param}}\left(x\right)=F\left(x_{1},x_{2}\ldots x_{i}\right)=y$$

where x_*i*_ is the vector of the *i*-th instance in the bag; *n* is the number of instances in the bag; and *F*(·) is the fixed aggregation function that computes the pooling output of the bag, y.

Parameterized pooling methods, on the other hand, have learnable parameters. These parameters are adjusted during the learning process to better capture the characteristics of the bags. One example is self-attention pooling, in which the attention scores are computed using a set of learnable parameters (the weight matrices that project input to queries, keys, and values).^46^^,^^47^ They can be expressed as follows:

(2) $$f_{\text{param}}\left(x\right)=\sum_{i=1}^{n}\alpha_{i}F_{\text{proj}}\left(x_{i}\right)=y$$

where *x*_*i*_ is the vector of the *i*-th instance in the bag; *n* is the number of instances in the bag; *α*_*i*_ is a weight learned during the training process that determines the contribution of instance *i*'s projection *F*_proj_(x_*i*_) to the overall pooling output, y.

Average pooling (AP)^48^ and Hopfield pooling (HP)^49^ were used as aggregation methods to represent nonparameterized pooling and parameterized pooling, respectively. The aim of the study was to demonstrate the generalizability of comparing these two aggregation methods, regardless of the aggregation methods chosen. HP's weights were trained on PyTorch Metric Learning^50^ using the MetricLossOnly trainer with supervised contrastive loss.^51^

### WSI Retrieval

The process of creating WSI-level embedding is essentially a transformation that converts high-dimensional data with redundant, irrelevant, and noisy information from many patches into a single compact numeric representation. Thus, the WSI and its embedding have a one-to-one mapping relationship. By using nearest neighbor algorithms on these embeddings, semantically similar instances can be retrieved, a process known as vector search.^52^ Modern image search engines such as Yottixel use a binarized vector based on Min-Max algorithm to expedite retrieval.^19^^,^^53^^,^^54^

To retrieve WSIs, the distances between an input query WSI and all WSIs in the database were calculated, ranked in ascending order, and the top-*n* results returned. The performance of the WSI retrieval task using the mean average precision at *n* (mAP@n) metric with *n* = 10 was evaluated.^55^ For a single WSI query, the mean average precision can be given as follows:

(3) $$\text{mAP}@n=\frac{1}{n}\sum_{i=1}^{10}P\left(i\right)$$

(4) $$P\left(i\right)=\left\{ \begin{array}{c} \frac{\text{TP}}{\text{TP}+\text{FP}},\;\text{if}\;\text{the}\;i-\text{th}\;\text{retrieval}\;\text{is}\;\text{correct}\\ \;0,\;\text{otherwise} \end{array}\right.$$

where true positive (TP) is the number of retrieved WSIs that share the same label as the query, and false positive (FP) is the number of retrieved WSIs that do not have the same label.

The performances were compared with and without using the BPG step as performance improvements and used a one-tailed test to determine if the improvements were significant.

The mean of improvements across eight distinct settings, including combinations of pooling methods (eg, average pooling and Hopfield pooling) and feature extraction methods (eg, ViT-16/256, KimiaNet, DenseNet, and DINO) was used to represent the overall improvement effect of BPG.

### Slide-Level Predictions by *k*-Nearest Neighbor

In the pipeline, to predict the WSI label from slide representations, a weighted *k*-nearest neighbor classifier optimized through grid search with cross-validation was used, selecting the optimal *k* value from the range [1, 3, 5, …, 19] (the range of *k* is heuristically determined as from 1 to the nearest odd number of the reference data set size's square root, 19). The cosine distance for the query vector v_*q*_ and the any other WSI representation v_*p*_ is defined as follows:

(5) $$\text{cosine}\;\text{distance}=D_{C}\left(v_{q},v_{p}\right):=1-S_{C}\left(v_{q},v_{p}\right)=1-\frac{\sum_{i=1}^{n}v_{q}\left(i\right)v_{p}\left(i\right)}{\sqrt{\sum_{i=1}^{n}v_{q}{\left(i\right)}^{2}}\sqrt{\sum_{i=1}^{n}v_{p}{\left(i\right)}^{2}}}$$

The *k*-nearest neighbor is a classification method that assigns an instance to the class most prevalent among its *k* nearest neighbors that sometimes could be determined by a majority vote. In weighted *k*-nearest neighbor,^56^ the *k* closest neighbors are assigned different weights based on their distance to the query point, with closer neighbors having a greater influence on the output. Unlike model-based algorithms, this is an instance-based method that does not learn weights from training data and is used commonly to evaluate embeddings with an easy-to-understand prediction scheme.^55^ The study used *k*-nearest neighbor, which is implemented in Python's Scikit-learn package.^57^

Weighted-average F1 score (weighted-F1) (ie, the F1 score of all classes' aggregated contributions) was used because the data set was imbalanced (Supplemental Table S1) and more weight should be given to classes with larger examples, to represent the classification performance. Weighted F1 is defined as follows:

(6) $$\begin{array}{c} \text{precisio}n_{i}=\frac{TP_{i}}{TP_{i}+FP_{i}}\\ \text{recal}l_{i}=\frac{TP_{i}}{TP_{i}+FN_{i}}\\ F1_{i}=2\times \frac{\text{precisio}n_{i}\times \text{recal}l_{i}}{\text{precisio}n_{i}+\text{recal}l_{i}}\\ F1_{\text{weighted}}=\frac{\sum_{i=1}^{C}w_{i}\times \text{F}1_{i}}{\sum_{i=1}^{C}w_{i}} \end{array}$$

where *C* is the number of classes, F1_*i*_ is the F1 score for class *i*, and w_*i*_ is the weight assigned to class *i*.

Performances with and without using the BPG step as performance improvements were compared and a one-tailed test was used to determine whether the improvements were significant.

The mean of improvements across eight distinct settings, including combinations of pooling methods (eg, average pooling and Hopfield pooling) and feature extraction methods (eg, ViT-16/256, KimiaNet, DenseNet, and DINO), was used to represent the overall improvement effect of BPG.

### Code Availability

The deep models used are all available online. The k-means algorithm used was taken from Python's Scikit-learn package.^57^ The code for this system is available at *https://zenodo.org/record/10484495*.

## Results

### Processed Bag Comparison

A bone marrow trephine core biopsy specimen WSI data set was used to investigate the effect of BPG on WSI representation quality. Bone marrow biopsy specimens provide a logical test case because they may be resolved easily into tissue (diagnostically relevant) and nontissue (not diagnostically relevant) regions. Therefore, the effect of BPG could be evaluated easily and rapidly in a binary-like manner by pathologists. As a first step, BPG was applied (see BPG) on the patch features from different feature extractors (see Feature Extractors). With BPG, the aim was to filter out irrelevant patches, keeping only those that are more likely to be from ROIs, as would be selected by a pathologist.

**Figure 2:**
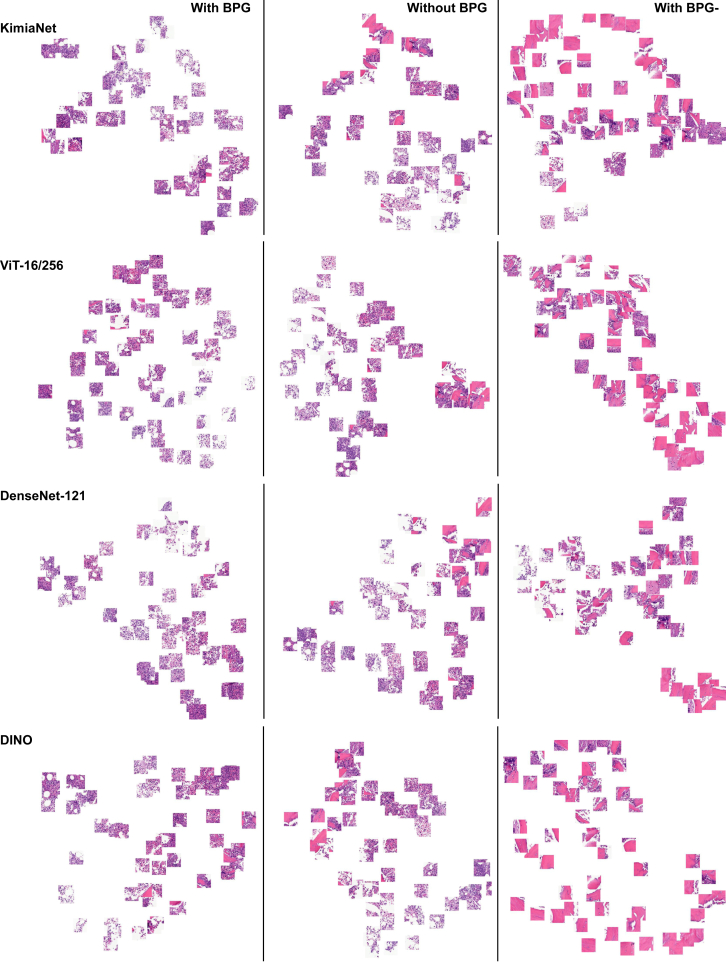
Comparison of patch samples under different clustering settings for different deep models. Patches selected through binary patch grouping (BPG) are predominantly the region of interest in the whole slide image. Additionally, the patches filtered out through BPG (BPG-) are mostly images of bone and muscle tissue. The locations of the patches are determined based on the Uniform Manifold Approximation and Projection projections of their features. Original magnification, ×40.

Uniform Manifold Approximation and Projection (UMAP) is a dimensionality reduction algorithm that allows visualization of a model's high-dimensional feature embedding space.^58^ To visually and qualitatively assess the effect of BPG on the embedding space of each feature extractor model, patch distribution with UMAP was visualized. When visualizing the patches from “with BPG,” “without BPG,” and “with BPG-,” it was clear that patches from “with BPG” were more representative of the ROIs in the WSI. The patches included areas that contained informative structures, such as the nuclei and tissue. On the other hand, the patches selected without BPG included a significant proportion of diagnostically irrelevant patches including bone, muscle, and hemorrhage. Moreover, the patches from BPG- almost entirely represented these irrelevant tissue structures (Figure 2). This suggested a feature extractor embedding space more representative of diagnostically meaningful patches.

### WSI Comparisons

**Figure 3:**
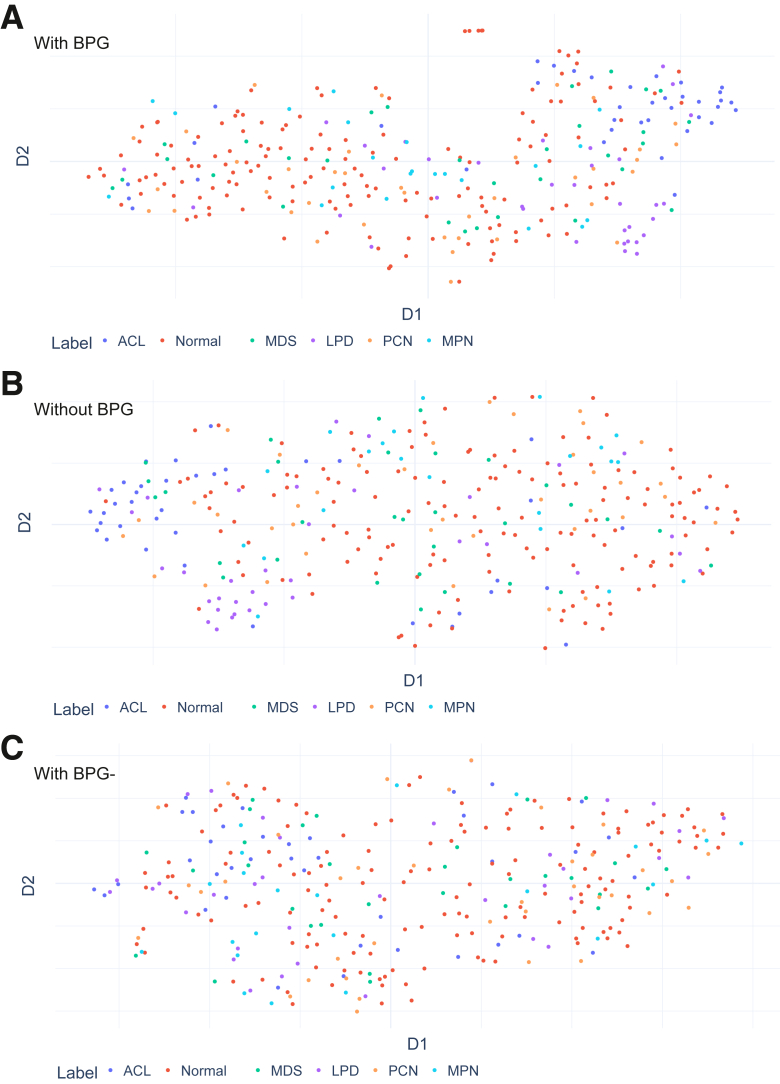
Visual comparison of slide-level representations in the test set through Uniform Manifold Approximation and Projection (UMAP). **A:** The UMAP projections of slide-level representations generated from patches selected through binary patch grouping (BPG) show a distinct clustering pattern, with the acute leukemia (ACL) clustering grouping together. **B:** In contrast, the UMAP projections of slide-level representations generated without using BPG show a less-pronounced clustering pattern, with the ACL cluster not grouping distinctly. **C:** Similarly, the UMAP projections of slide-level representations generated by BPG- are more spread out and have almost no evident clustering pattern. The representations from a single experiment with DINO as the feature extractor are used as examples. D, dimension; LPD, lymphoproliferative disorder; MDS, myelodysplastic syndrome; MPN, myeloproliferative neoplasm; PCN, plasma cell neoplasm.

Next, two feature aggregation methods were applied to the embedding space of each feature extractor model as outlined in Aggregation Methods and Figure 1. To visualize the bone marrow biopsy slide-level representations, UMAP dimensionality reduction was again applied to project the high-dimensional WSI representations into a two-dimensional space. When visualizing the UMAP projections of the slide-level representations generated from the patches selected by BPG, it was clear that they displayed a grouping pattern compared with non-BPG or BPG-. For example, the slide-level representations of acute leukemia cluster loosely together and provide some separation from the projections of other classes (Figure 3A). This made conceptual sense because acute leukemia is a binary diagnostic category in hematopathology and therefore provides a simple but effective scenario for evaluation.

On the other hand, the UMAP projections of the slide-level representations generated without BPG exhibited a less-obvious grouping pattern. The slide-level representations of acute leukemia did not tend to cluster (ie, were well separated), and mixed with projections from other classes (Figure 3B). Similarly, the UMAP projections of the slide-level representations generated from BPG- were more spread out and had almost no grouping pattern (Figure 3C).

### WSI Retrieval and Classification Comparison

Next, task evaluation was used to assess WSI representation quality in the context of BPG, and each feature extraction and aggregation method. The tasks of WSI retrieval and WSI classification are well-described metrics of slide-level representation quality in computer vision, and, in particular, the pathology domain.

First, experiments to conduct WSI retrievals were performed using the slide-level representations as features (see WSI Retrieval) in which the goal was to retrieve the most similar images (ie, images with the same diagnostic labels), given a query WSI (ie, slide-level feature vector). The performance of the approach using BPG with a baseline approach that did not use BPG, and another approach that used BPG-, were compared.

**Figure 4:**
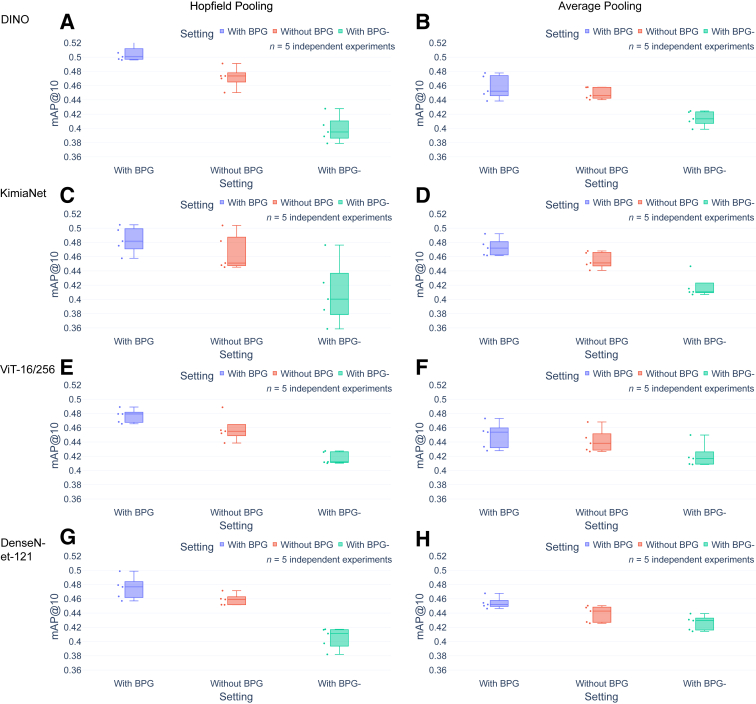
Comparison of whole slide image retrieval task performance using different approaches. **A**–**H:** The results of the evaluation show that the approach using binary patch grouping (BPG) achieved a higher mean average precision at 10 (mAP@10) score than the baseline approach and the approach using BPG-, regardless of feature extractors and aggregation methods. The mAP@10 score for the BPG approach was approximately 4% higher than the baseline approach, and approximately 15% higher than the approach using BPG-. Except for HIPT+average pooling (**D**), all differences between with and without BPG were statistically significant.

In general, the approach using BPG achieved a higher mAP score than the baseline approach and the approach using the BPG-, regardless of the selection of feature extractors and aggregation methods. The mAP score for BPG approach was approximately 0.02 better than the baseline approach (4% improvement), and approximately 0.06 better than the approach using BPG- (14% improvement) (Figure 4 and Supplemental Table S2). The effect of adding BPG at baseline is nearly equivalent to changing the aggregation method from AP to HP aggregation methods. Meanwhile, the more sophisticated aggregation method of HP produced better results than simple AP when keeping the feature extractors the same. With BPG and HP, mAPDINO > mAPKimiaNet > mAPHIPT > mAPDenseNet-121 was observed. That mAPDenseNet-121 is the lowest may be expected because DenseNet-121 is a convolution-based network and has been trained on ImageNet. In contrast, mAP_KimiaNet_ > mAP_HIPT_ was surprising because KimiaNet is a retrained convolution model at ×20 magnification, whereas HIPT is an attention-based model trained at several scales/magnifications on pathology WSI. mAP_DINO_ was the highest, which also was not expected because it was trained on ImageNet.

In addition to search and retrieval, a WSI classification task was performed using the slide-level representations as features (see Slide-Level Predictions by k-Nearest Neighbor), in which the goal was to classify the WSIs into six classes (simplified slide-level diagnostic labels, see Data Set). The performance of the approach using BPG with a baseline approach that did not use BPG, and another approach that used BPG-, were compared.

**Figure 5:**
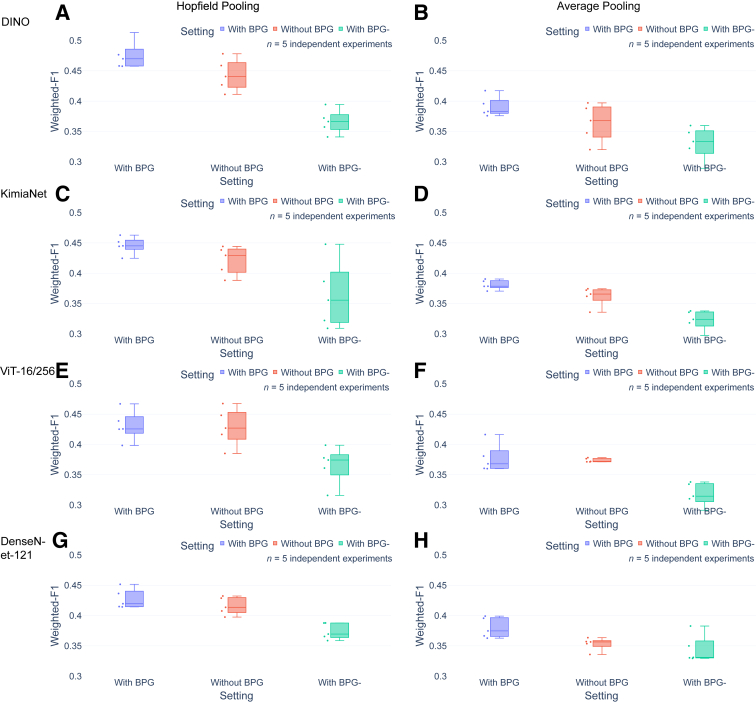
Comparison of whole slide image classification task performance using different approaches. **A**–**H:** The results of the evaluation show that the approach using binary patch grouping (BPG) achieved a higher weighted-F1 score than the baseline approach and the approach using BPG-. The weighted-F1 score for the BPG approach was approximately 5% higher than the baseline approach, and 20% higher than the approach using BPG-. **B, C,** and **F:** Except for DINO+average pooling (AP) (**B**), HIPT+Hopfield pooling (HP) (**C**), and DenseNet-121+AP (**F**), all differences between with and without BPG were statistically significant. However, the effect was less than changing the aggregation method from HP to AP. For the comparison in recall and precision, see Supplemental Tables S4 and S5.

The use of BPG achieved a higher weighted-F1 score than the baseline approach and the approach using BPG-. The weighted-F1 score for the BPG approach was approximately 0.02 higher than the baseline approach (5% relative improvement), and 0.06 higher than the BPG-relative approach (18% improvement) (Figure 5 and Supplemental Tables S3–S5).

With BPG and HP, weighted-F1_DINO_ > weighted-F1_KimiaNet_ > weighted-F1_HIPT_ > weighted-F1_DenseNet-121_ was observed. Weighted-F1_DenseNet-121_ had the lowest classification accuracy, which was expected for the retrieval task as detailed in the preceding paragraphs. In contrast, weighted-F1_KimiaNet_ > weighted-F1_HIPT_ again was a surprise for classification because KimiaNet is a retrained convolution model at ×20 whereas HIPT is an attention-based model trained at several scales/magnifications. That weighted-F1_DINO_ had the highest classification accuracy also was not expected because it is trained on ImageNet, albeit with an attention-based, self-supervised approach.

### Optimal Pipeline: DINO+BPG+HP

In the context of the findings from this study, the optimal pipeline for slide-level representation among those evaluated would involve using DINO+BPG+HP. The parameterized (attention-based) pooling technique, HP, can be viewed as a function that extracts pathology-specific features from patch feature bags while aggregating them into a single slide-level vector. This method was expected to produce more favorable results than a simple, nonparameterized pooling method. When HP is applied, the process is a typical transfer learning in which a second model (HP) learns task-specific features from the first model's lower-level features (bags).^59^ Thus, the advantages of DINO+HP may be explained in the context of DG versus DS pretrained models in transfer learning.

As mentioned in Feature Extractors, DG large-model trained with SSL may outperform DS models in transfer learning because these apparently generate richer features for downstream models and are less prone to overfitting. This is supported by the results from this study, which showed that DINO with HP outperformed the other models in both WSI retrieval and WSI classification tasks (Table 1).

**Table 1:** Comparison of Pretrained Models for WSI Retrieval and Classification Tasks When BPG and Hopfield Pooling Were Used

Model	mAP@10	Weighted-F1
DINO	0.506 ± 0.0144	0.475 ± 0.023
KimiaNet	0.483 ± 0.019	0.446 ± 0.020
HIPTViT-16/256	0.476 ± 0.010	0.431 ± 0.012
DenseNet-121	0.475 ± 0.015	0.427 ± 0.008

Results show that DINO, a vision transformer (large modal) trained on ImageNet (large nonmedical data set) using self-supervised learning, outperforms other models. Specifically, DINO achieves a 0.02 better mean average precision at 10 (mAP@10) and 0.03 better weighted-F1 compared with KimiaNet, a 0.03 higher mAP@10 and 0.04 higher weighted-F1 compared with HIPT, and a 0.03 higher mAP@10 and 0.05 higher weighted-F1 compared with DenseNet-121 (one-tailed *P* < 0.05, DINO versus the other models).

BPG, binary patch grouping; WSI, whole slide image.

To further examine the richness of information captured by each pretrained model, principal component analysis was applied to the patch features of the Profiling Data Set (generated for training BPG, see BPG) to determine the number of components needed to explain 95% of the variance. HIPT_ViT-16/256_ feature vectors of size 384 components could be reduced to just 11 components, which was 2.9% of the original feature size, whereas KimiaNet features could be reduced to 8.2%, DenseNet-121 features could be reduced to 28.6%, and DINO's features could be reduced to 53.3% (Table 2). These ratios suggest that the large model, HIPT_ViT-16/256_, may have overfitted to its DS training data set of The Cancer Genome Atlas. Overall, parameterized pooling with DG feature extraction produced superior results compared with other combinations.

**Table 2:** Feature Variability Comparison Using Principal Component Analysis

Model	Components explaining 95% of variance, n	Original feature vector length	Ratio
DINO (DG, large)	205	384	≈53%
KimiaNet (DS, small)	84	1024	≈8%
HIPT_ViT-16/256_ (DS, large)	11	384	≈3%
DenseNet-121 (DG, small)	293	1024	≈29%

The results show that features from domain-specific (DS) models (namely, KimiaNet and HIPT) have lower variability compared with their domain-general (DG) counterparts (namely, DenseNet-121 and DINO). This suggests that the DS models may show less flexibility compared with DG models to fit unseen data.

## Discussion

The findings from this study show that in bone marrow histopathology, a simple, pathologist-centered intervention step, BPG, improved WSI representation quality. In addition, DG models, specifically DenseNet-121 and DINO, showed unexpected success as feature extractors in the WSI representation pipeline. DenseNet-121 performed comparably with the DS model HIPT in the WSI retrieval task, whereas DINO outperformed all other models when applied with BPG and HP. The limited improvement of DS to DG was similar to one study that found fine-tuning feature extractors on histopathology images only increased the area under the curve in the gastrointestinal data set from 91.1 to 92.9.^37^ Likewise, as reported from HIPT, ViT-16/256 did not outperform the ImageNet-trained ResNet-50 (truncated after the third residual block) in some of the downstream tasks.^42^ The difference between the primary bone marrow data set and The Cancer Genome Atlas data set used by HIPT and KimiaNet was suspected to have limited the benefits of these DS models, which did not include bone marrow histopathology in their training sets. On the other hand, DG models (DINO and DenseNet-121) appeared to extract more diverse features from the patches than DS models (see Optimal Pipeline: DINO+BPG+HP), including a larger spectrum of noise and signals (in terms of histopathology). As HP learns to assign more weight to signals than noise using an attention mechanism (prioritizing and deprioritizing certain data elements),^60^ this method is expected to work better when more signal features are preserved. Therefore, DINO's superiority appeared when HP was used (Supplemental Tables S2 and S3). Overall, DS training made the model more specialized and trimmed down domain-irrelevant information (Table 2). However, this also made the model more sensitive to changes in input conditions, such as lighting, stain quality, or even small changes to WSI class in the same domain (eg, bone marrow versus other pathology tissue site), resulting in the disadvantage of HIPT against DINO. A large model trained on a large data set using SSL such as DINO, appears to be more suitable for being a baseline feature extractor when transfer learning is applied.

In general, removing irrelevant patches, as in the case of BPG, improved the quality of slide-level representations and helped the downstream computational pathology tasks, such as WSI classification and interslide WSI search, which was measured by using weighted-F1 and mAP@10. Weighted-F1 shows the quality of WSI representation in classification tasks, whereas mAP@10 considers the ranking of relevant items within the top 10 positions. Unlike weighted-F1, which focuses on overall classification accuracy, mAP@10 provides a nuanced understanding of the model's ability to prioritize and rank similar items in retrieval settings. The combination of weighted-F1 and mAP@10 offers a more comprehensive evaluation of WSI representation quality. The simplicity of the clustering step and the minimum human intervention may justify using the proposed scheme compared with more complex frameworks. Furthermore, it is pathologist-centric, allowing the pathologist to provide minimum critical input in the WSI representation pipeline. The improvements varied depending on the training task. For WSI retrieval, the BPG approach relatively increased the performance by 4%, similar to switching from AP to HP. However, for the classification task, the BPG approach relatively boosted the performance by approximately 5%, but the effect was less than changing the aggregation method from AP to HP. Similar to other information-refining processes, useful signals (ie, some diagnostically relevant patches), mistakenly might be excluded. However, as long as the grouped patches collectively hold sufficient diagnostic relevance, even if individual diagnostically relevant patches occasionally are excluded, the overall quality of the final slide-level representation remains robust, which is supported by the performance improvement.

It certainly is plausible that the effectiveness of the BPG step is dependent on the expertise of the pathologist as well as the pathology domain. Input from one hematopathologist was attained in this proof-of-concept study. To reduce the potential for individual human error and enhance the reliability of the results, involving a panel of pathologists to make decisions collectively during the BPG step would be ideal. Still, the ability to function independently of pathologists, or using fewer pathologists, would make the pipeline more valuable. The reliance on a single data set without external validation raises the possibility that the binary nature of BPG may not be applicable universally, particularly in more complex tissue specimens. Generally, bone marrow tissue has very well-defined relevant and nonrelevant patches compared with other pathology tissue sites. As a result, this method may be restricted to bone marrow. Exploring its adaptability in more complex tissue specimens across diverse histopathologic scenarios will be important for future research in expanding the applicability of BPG.

Deliberately selecting binary clustering with k = 2 in the k-means algorithm serves a specific purpose: discerning between noise and signal groups, which is particularly relevant to bone marrow, which has quite distinct relevant and nonrelevant tissue patches. Opting for additional clusters may not necessarily enhance the separation, however, conversely, may affect the quality of the training data detrimentally. As the number of clusters increases, patches along the clustering edges that should belong to the signal group are more likely to be grouped erroneously into noise clusters. Thus, the intentional choice of k = 2 aims to optimize the differentiation between noise and signal while minimizing the risk of misclassification along the clustering edges.

Overall, the current results demonstrated that the improvement shown by BPG holds regardless of the choice of feature extractor and aggregation methods. BPG is an adaptable plugin that can be integrated into various pipelines to enhance the quality of slide-level representation, which focuses on improving existing processes rather than replacing them. Overall, the findings may have relevance to the design of computational pathology software to support WSI representation in digital pathology. Further studies on larger and more general pathology WSI data sets will be informative. Future studies ideally would evaluate scalability with larger data sets and more diverse pathologist input.

## Author Contributions

H.R.T., T.D., S.A., and C.J.V.C. conceptualized the study; Y.M., H.R.T., and C.J.V.C. designed the experiments; Y.M., H.R.T., C.J.V.C., T.D., and S.A. analyzed the data; Y.M. performed the experiments and developed the software; and Y.M., H.R.T., and C.J.V.C. wrote the manuscript.

## Disclosure Statement

None declared.

## References

[1] Kumar V., Abbas A.K., Fausto N., Aster J.C. (2014). Professional Edition E-Book.

[2] Janowczyk A., Madabhushi A. (2016). Deep learning for digital pathology image analysis: a comprehensive tutorial with selected use cases. J Pathol Inform.

[3] Echle A., Rindtorff N.T., Brinker T.J., Luedde T., Pearson A.T., Kather J.N. (2021). Deep learning in cancer pathology: a new generation of clinical biomarkers. Br J Cancer.

[4] Tran K.A., Kondrashova O., Bradley A., Williams E.D., Pearson J.V., Waddell N. (2021). Deep learning in cancer diagnosis, prognosis and treatment selection. Genome Med.

[5] Jiang Y., Yang M., Wang S., Li X., Sun Y. (2020). Emerging role of deep learning–based artificial intelligence in tumor pathology. Cancer Commun.

[6] Kumar Y., Gupta S., Singla R., Hu Y.-C. (2022). A systematic review of artificial intelligence techniques in cancer prediction and diagnosis. Arch Comput Methods Eng.

[7] Tizhoosh H.R., Pantanowitz L. (2018). Artificial intelligence and digital pathology: challenges and opportunities. J Pathol Inform.

[8] Khened M., Kori A., Rajkumar H., Krishnamurthi G., Srinivasan B. (2021). A generalized deep learning framework for whole-slide image segmentation and analysis. Sci Rep.

[9] Tang Z., Chuang K.V., DeCarli C., Jin L.-W., Beckett L., Keiser M.J., Dugger B.N. (2019). Interpretable classification of Alzheimer's disease pathologies with a convolutional neural network pipeline. Nat Commun.

[10] Fan K., Wen S., Deng Z. (2019). Innovation in Medicine and Healthcare Systems, and Multimedia: Proceedings of KES-InMed-19 and KES-IIMSS-19 Conferences.

[11] Xu J., Luo X., Wang G., Gilmore H., Madabhushi A. (2016). A deep convolutional neural network for segmenting and classifying epithelial and stromal regions in histopathological images. Neurocomputing.

[12] Tosun A.B., Nguyen L., Ong N., Navolotskaia O., Carter G., Fine J.L., Taylor D.L., Chennubhotla S.C. (2017). Medical Image Computing and Computer-Assisted Intervention—MICCAI 2017: 20th International Conference, Quebec City, QC, Canada, September 11-13, 2017, Proceedings, Part II..

[13] Tavolara T.E., Gurcan M.N., Niazi M.K.K. (2022). Contrastive multiple instance learning: an unsupervised framework for learning slide-level representations of whole slide histopathology images without labels. Cancers (Basel).

[14] Guan Y., Zhang J., Tian K., Yang S., Dong P., Xiang J., Yang W., Huang J., Zhang Y., Han X. (2022). Node-aligned graph convolutional network for whole-slide image representation and classification. Proceedings of the IEEE/CVF Conference on Computer Vision and Pattern Recognition. New Orleans, LA.

[15] Sharma Y., Shrivastava A., Ehsan L., Moskaluk C.A., Syed S., Brown D. (2021). Medical Imaging with Deep Learning.

[16] Hemati S., Kalra S., Babaie M., Tizhoosh H.R. (2023). Learning binary and sparse permutation-invariant representations for fast and memory efficient whole slide image search. Comput Biol Med.

[17] Mikolov T., Chen K., Corrado G., Dean J. (2013). Efficient estimation of word representations in vector space. arXiv.

[18] Campanella G., Hanna M.G., Geneslaw L., Miraflor A., Werneck V., Silva K., Busam K.J., Brogi E., Reuter V.E., Klimstra D.S., Fuchs T.J. (2019). Clinical-grade computational pathology using weakly supervised deep learning on whole slide images. Nat Med.

[19] Kalra S., Tizhoosh H.R., Choi C., Shah S., Diamandis P., Campbell C.J.V., Pantanowitz L. (2020). Yottixel–an image search engine for large archives of histopathology whole slide images. Med Image Anal.

[20] Tsai P.-C., Lee T.-H., Kuo K.-C., Su F.-Y., Lee T.-L.M., Marostica E., Ugai T., Zhao M., Lau M.C., Väyrynen J.P., Giannakis M. (2023). Histopathology images predict multi-omics aberrations and prognoses in colorectal cancer patients. Nat Commun.

[21] Kalra S., Tizhoosh H.R., Shah S., Choi C., Damaskinos S., Safarpoor A., Shafiei S., Babaie M., Diamandis P., Campbell C.J.V., Pantanowitz L. (2020). Pan-cancer diagnostic consensus through searching archival histopathology images using artificial intelligence. NPJ Digit Med.

[22] Huang G., Liu Z., Van Der Maaten L., Weinberger K.Q. (2017). Proceedings of the IEEE Conference on Computer Vision and Pattern Recognition.

[23] He K., Zhang X., Ren S., Sun J. (2016). Proceedings of the IEEE Conference on Computer Vision and Pattern Recognition.

[24] Gildenblat J., Ben-Shaul I., Lapp Z., Klaiman E. (2021). Pattern Recognition. ICPR International Workshops and Challenges. Virtual Event, January 10–15, 2021, Proceedings, Part I..

[25] Yao J., Zhu X., Jonnagaddala J., Hawkins N., Huang J. (2020). Whole slide images based cancer survival prediction using attention guided deep multiple instance learning networks. Med Image Anal.

[26] Bidgoli A.A., Rahnamayan S., Dehkharghanian T., Riasatian A., Tizhoosh H.R. (2023). Evolutionary computation in action: hyperdimensional deep embedding spaces of gigapixel pathology images. IEEE Trans Evol Comput.

[27] Lloyd S. (1982). Least squares quantization in pcm. IEEE Trans Inf Theory.

[28] Ikotun A.M., Ezugwu A.E., Abualigah L., Abuhaija B., Heming J. (2023). K-means clustering algorithms: a comprehensive review, variants analysis, and advances in the era of big data. Inf Sci.

[29] Ciga O., Xu T., Nofech-Mozes S., Noy S., Lu F.-I., Martel A.L. (2021). Overcoming the limitations of patch-based learning to detect cancer in whole slide images. Sci Rep.

[30] Jiao Y., Li J., Fei S. (2022). Staining condition visualization in digital histopathological whole-slide images. Multimed Tool Appl.

[31] Hassan M., Mollick S., Yasmin F. (2022). An unsupervised cluster-based feature grouping model for early diabetes detection. Healthcare Anal.

[32] Caron M., Touvron H., Misra I., Jégou H., Mairal J., Bojanowski P., Joulin A. (2021). Emerging properties in self-supervised vision transformers. Proceedings of the IEEE/CVF International Conference on Computer Vision and Pattern Recognition. Nashville, TN.

[33] Mu Y., Tizhoosh H.R., Tayebi R.M., Ross C., Sur M., Leber B., Campbell C.J.V. (2021). A BERT model generates diagnostically relevant semantic embeddings from pathology synopses with active learning. Commun Med.

[34] Xu Q.-S., Liang Y.-Z. (2001). Monte Carlo cross validation. Chemometr Intell Lab Syst.

[35] Deng J., Dong W., Socher R., Li L.-J., Li K., Fei-Fei L. (2009). 2009 IEEE Conference on Computer Vision and Pattern Recognition.

[36] Cooper L., Demicco E.G., Saltz J.H., Powell R.T., Rao A., Lazar A.J. (2018). Pancancer insights from The Cancer Genome Atlas: the pathologist's perspective. J Pathol.

[37] Sharmay Y., Ehsany L., Syed S., Brown D.E. (2021). 2021 IEEE EMBS International Conference on Biomedical and Health Informatics (BHI).

[38] Riasatian A., Babaie M., Maleki D., Kalra S., Valipour M., Hemati S., Zaveri M., Safarpoor A., Shafiei S., Afshari M., Rasoolijaberi M. (2021). Fine-tuning and training of densenet for histopathology image representation using TCGA diagnostic slides. Med Image Anal.

[39] He K., Fan H., Wu Y., Xie S., Girshick R. (2020). Proceedings of the IEEE/CVF Conference on Computer Vision and Pattern Recognition.

[40] Chen T., Kornblith S., Norouzi M., Hinton G. (2020). A. simple framework for contrastive learning of visual representations. arXiv.

[41] Yang X., He X., Liang Y., Yang Y., Zhang S., Xie P. (2020). Transfer learning or self-supervised learning? A tale of two pretraining paradigms. arXiv.

[42] Chen R.J., Chen C., Li Y., Chen T.Y., Trister A.D., Krishnan R.G., Mahmood F. (2022). Proceedings of the IEEE/CVF Conference on Computer Vision and Pattern Recognition.

[43] Dosovitskiy A., Beyer L., Kolesnikov A., Weissenborn D., Zhai X., Unterthiner T., Dehghani M., Minderer M., Heigold G., Gelly S., Uszkoreit J., Houlsby N. (2020). An image is worth 16x16 words: transformers for image recognition at scale. arXiv.

[44] Wang X., Yan Y., Tang P., Liu W., Guo X. (2019). Bag similarity network for deep multi-instance learning. Inf Sci.

[45] Xiong D., Zhang Z., Wang T., Wang X. (2021). A comparative study of multiple instance learning methods for cancer detection using T-cell receptor sequences. Comput Struct Biotechnol J.

[46] Vaswani A., Shazeer N., Parmar N., Uszkoreit J., Jones L., Gomez A.N., Kaiser T., Polosukhin I. (2017). Attention is all you need. arXiv.

[47] Safari P., India M., Hernando J. (2020). Self-attention encoding and pooling for speaker recognition. arXiv.

[48] Boureau Y.-L., Ponce J., LeCun Y. (2010). IEEE Computer Society Conference on Computer Vision and Pattern Recognition.

[49] Ramsauer H., Schäfl B., Lehner J., Seidl P., Widrich M., Adler T., Gruber L., Holzleitner M., Pavlovic M., Sandve G.K., Greiff V., Kreil D., Kopp M., Klambauer G., Brandstetter J., Hochreiter S. (2020). Hopfield networks is all you need. arXiv.

[50] Musgrave K., Belongie S., Lim S.-N. (2020). PyTorch metric learning. arXiv.

[51] Khosla P., Teterwak P., Wang C., Sarna A., Tian Y., Isola P., Maschinot A., Liu C., Krishnan D. (2020). Supervised contrastive learning. Adv Neural Inf Process Syst.

[52] Platzer C., Dustdar S. (2005). Third European Conference on Web Services (ECOWS’05).

[53] Tizhoosh H.R., Zhu S., Lo H., Chaudhari V., Mehdi T. (2016). MinMax radon barcodes for medical image retrieval. arXiv.

[54] Kumar M.D., Babaie M., Tizhoosh H.R. (2018). 2018 International Joint Conference on Neural Networks (IJCNN).

[55] Musgrave K., Belongie S., Lim S.-N. (2020). European Conference on Computer Vision.

[56] Fix E., Hodges J.L. (1989,). Discriminatory analysis. nonparametric discrimination: consistency properties. Int Stat Rev.

[57] Pedregosa F., Varoquaux G., Gramfort A., Michel V., Thirion B., Grisel O., Blondel M., Prettenhofer P., Weiss R., Dubourg V., Vanderplas J., Passos A., Cournapeau D., Brucher M., Perrot M., Duchesnay E. (2011). Scikit-learn: machine learning in Python. J Mach Learn Res.

[58] McInnes L., Healy J., Melville J. (2018). UMAP: Uniform Manifold Approximation and Projection for dimension reduction. arXiv.

[59] Pan S.J., Yang Q. (2010). A survey on transfer learning. IEEE Trans Knowl Data Eng.

[60] Guo M.-H., Xu T.-X., Liu J.-J., Liu Z.-N., Jiang P.-T., Mu T.-J., Zhang S.-H., Martin R.R., Cheng M.-M., Hu S.-M. (2022). Attention mechanisms in computer vision: a survey. Comput Vis Media.

