# Supplementary material for: Model-Agnostic Binary Patch Grouping for Bone Marrow Whole Slide Image Representation
Source: Am J Pathol. 2024 Feb 5;194(5):721–34. doi: 10.1016/j.ajpath.2024.01.012 (PMC12178382; doi:10.1016/j.ajpath.2024.01.012)
Supplement: Supplemental Table S1 [file mmc1.docx]

Supplemental Table S1: The count of labels.

| label | count |
| --- | --- |
| NORMAL | 305 |
| PCN | 76 |
| ACL | 75 |
| LPD | 69 |
| MDS | 63 |
| MPN | 45 |
