# Supplementary material for: Model-Agnostic Binary Patch Grouping for Bone Marrow Whole Slide Image Representation
Source: Am J Pathol. 2024 Feb 5;194(5):721–34. doi: 10.1016/j.ajpath.2024.01.012 (PMC12178382; doi:10.1016/j.ajpath.2024.01.012)
Supplement: Supplemental Table S2 [file mmc2.docx]

Supplemental Table S2: The mAP@10 comparison across different training settings. We generally observed mAP${}_{\text{DINO}}>$mAP${}_{\text{KimiaNet}}>$mAP${}_{\text{HIPT}}>$mAP${}_{\text{DenseNet}}$. Excluding the less relevant patch cluster through BPG approach provides the highest mAP values for all models used. Aggregating selected patch feature vectors achieved a 4% boost of mAP@10 score on the baseline approach (7 out of 8 differences were statistically significant, a notable improvement in the WSI retrieval task after applying BPG) and a 14% boost on the approach using the BPG-. This result is comparable to changing the aggregation method from AP to HP. (*:one-tailed p-value < 0.05, With BPG vs. Without BPG)

| Extraction | Setting  Agg Method | With BPG | Without BPG | With BPG- |
| --- | --- | --- | --- | --- |
| DINO | HP | 0.506±0.014* | 0.472±0.015 | 0.399±0.019 |
|  | AP | 0.458±0.017* | 0.449±0.008 | 0.414±0.010 |
| KimiaNet | HP | 0.483±0.019* | 0.466±0.026 | 0.409±0.044 |
|  | AP | 0.473±0.012* | 0.455±0.011 | 0.418±0.016 |
| HIPTViT-16/256 | HP | 0.476±0.010* | 0.458±0.018 | 0.417±0.008 |
|  | AP | 0.449±0.018 | 0.442±0.017 | 0.421±0.017 |
| DenseNet-121 | HP | 0.475±0.015* | 0.459±0.008 | 0.405±0.015 |
|  | AP | 0.454±0.008* | 0.439±0.011 | 0.426±0.010 |
| Random |  | 0.402±0.015 | 0.399±0.022 | 0.403±0.018 |
