# Supplementary material for: Model-Agnostic Binary Patch Grouping for Bone Marrow Whole Slide Image Representation
Source: Am J Pathol. 2024 Feb 5;194(5):721–34. doi: 10.1016/j.ajpath.2024.01.012 (PMC12178382; doi:10.1016/j.ajpath.2024.01.012)
Supplement: Supplemental Table S3 [file mmc3.docx]

Supplemental Table S3: The weighted-averaged F1 comparison across different training settings. we observed F1${}_{\text{DINO}}>$F1${}_{\text{KimiaNet}}>$F1${}_{\text{HIPT}}>$F1${}_{\text{DenseNet}}$. Aggregating the deep feature vectors of the selected BPG cluster provided the best results, achieving a 5% boost of F1 score on the baseline approach (6 out of 8 differences were statistically significant, a notable improvement in the WSI classification task after applying BPG) and an 19% boost on the approach using the BPG-. Although, the effect is less than changing the aggregation method from AP to HP. (*:one-tailed p-value < 0.05, With BPG vs. Without BPG)

| Extraction | Setting  Agg Method | With BPG | Without BPG | With BPG- |
| --- | --- | --- | --- | --- |
| DINO | HP | 0.475±0.023* | 0.443±0.026 | 0.366±0.020 |
|  | AP | 0.391±0.017* | 0.364±0.031 | 0.331±0.027 |
| KimiaNet | HP | 0.446±0.014* | 0.421±0.023 | 0.364±0.056 |
|  | AP | 0.381±0.008* | 0.362±0.015 | 0.323±0.016 |
| HIPTViT-16/256 | HP | 0.431±0.025 | 0.429±0.031 | 0.366±0.031 |
|  | AP | 0.377±0.023 | 0.374±0.003 | 0.318±0.019 |
| DenseNet-121 | HP | 0.427±0.016* | 0.416±0.015 | 0.374±0.013 |
|  | AP | 0.380±0.017* | 0.353±0.010 | 0.345±0.023 |
| Random |  | 0.322±0.007 | 0.315±0.012 | 0.316±0.008 |
