# Supplementary material for: Model-Agnostic Binary Patch Grouping for Bone Marrow Whole Slide Image Representation
Source: Am J Pathol. 2024 Feb 5;194(5):721–34. doi: 10.1016/j.ajpath.2024.01.012 (PMC12178382; doi:10.1016/j.ajpath.2024.01.012)
Supplement: Supplemental Table S4 [file mmc4.docx]

Supplemental Table S4: The recall comparison across different training settings.(*:one-tailed p-value < 0.05, With BPG vs. Without BPG)

| Extraction | Setting  Agg Method | With BPG | Without BPG | With BPG- |
| --- | --- | --- | --- | --- |
| DINO | HP | 0.482±0.022* | 0.452±0.033 | 0.360±0.015 |
|  | AP | 0.369±0.026 | 0.354±0.025 | 0.323±0.064 |
| KimiaNet | HP | 0.456±0.018 | 0.426±0.033 | 0.348±0.060 |
|  | AP | 0.378±0.019* | 0.349±0.012 | 0.321±0.024 |
| HIPTViT-16/256 | HP | 0.409±0.026 | 0.405±0.036 | 0.346±0.035 |
|  | AP | 0.354±0.053 | 0.353±0.007 | 0.313±0.046 |
| DenseNet-121 | HP | 0.440±0.011* | 0.425±0.020 | 0.381±0.016 |
|  | AP | 0.367±0.019* | 0.334±0.006 | 0.329±0.034 |
| Random |  | 0.298±0.031 | 0.289±0.015 | 0.278±0.028 |
