# Supplementary material for: Model-Agnostic Binary Patch Grouping for Bone Marrow Whole Slide Image Representation
Source: Am J Pathol. 2024 Feb 5;194(5):721–34. doi: 10.1016/j.ajpath.2024.01.012 (PMC12178382; doi:10.1016/j.ajpath.2024.01.012)
Supplement: Supplemental Table S5 [file mmc5.docx]

Supplemental Table S5: The precision comparison across different training settings. (*:one-tailed p-value < 0.05, With BPG vs. Without BPG)

| Extraction | Setting  Agg Method | With BPG | Without BPG | With BPG- |
| --- | --- | --- | --- | --- |
| DINO | HP | 0.483±0.029* | 0.445±0.021 | 0.384±0.026 |
|  | AP | 0.456±0.021* | 0.397±0.056 | 0.397±0.052 |
| KimiaNet | HP | 0.452±0.020* | 0.435±0.025 | 0.398±0.057 |
|  | AP | 0.475±0.010* | 0.404±0.042 | 0.399±0.062 |
| HIPTViT-16/256 | HP | 0.474±0.033 | 0.473±0.026 | 0.409±0.036 |
|  | AP | 0.473±0.027 | 0.446±0.021 | 0.426±0.076 |
| DenseNet-121 | HP | 0.437±0.021 | 0.430±0.013 | 0.384±0.018 |
|  | AP | 0.438±0.031 | 0.412±0.040 | 0.419±0.047 |
| Random |  | 0.432±0.024 | 0.401±0.041 | 0.395±0.072 |
